# A Simplified Method for Predicting Pattern Match Ratio

**DOI:** 10.3389/fpsyg.2021.704724

**Published:** 2021-09-03

**Authors:** Xiaojuan Tang, Huiqiong Duan, Shuliang Ding, Mengmeng Mao

**Affiliations:** ^1^School of Education, Jiangxi Normal University, Nanchang, China; ^2^School of Foreign Languages, Nanchang Hangkong University, Nanchang, China; ^3^School of Computer and Information Engineering, Jiangxi Normal University, Nanchang, China; ^4^School of Public Administration, Nanchang University, Nanchang, China

**Keywords:** cognitive diagnostic test design, pattern match ratio, theoretical construct validity, prediction method, upper bound

## Abstract

Cognitive diagnostic test design (CDTD) has a direct impact on the pattern match ratio (PMR) of the classification of examinees. It is more helpful to know the quality of a test during the stage of the test design than after the examination is taken. The theoretical construct validity (TCV) is an index of the test quality that can be calculated without testing, and the relationship between the PMR and the TCV will be revealed. The TCV captures the three aspects of the appeal of the test design as follows: (1) the TCV is a measure of test construct validity, and this index will navigate the processes of item construction and test design toward achieving the goal of measuring the intended objectives, (2) it is the upper bound of the PMR of the knowledge states of examinees, so it can predict the PMR, and (3) it can detect the defects of test design, revise the test in time, improve the efficiency of test design, and save the cost of test design. Furthermore, the TCV is related to the distribution of knowledge states and item categories and has nothing to do with the number of items.

## Introduction

Cognitive diagnosis (CD) has received much attention, providing diagnostic information of knowledge or skills (often called “attributes” in the CD literature) to the examinees (de la Torre and Douglas, 2004; de la Torre, 2008; DeCarlo, 2011; Liu et al., 2012; Kang et al., 2017; Huebner et al., 2018). It is critical to ensure that high-quality cognitive diagnostic tests can accurately diagnose the knowledge state (KS, i.e., the latent cognitive states) of examinees. The set of KSs is represented by the Q_S_ matrix. In fact, cognitive diagnostic test design (CDTD) is the design of a Q matrix, called Q_t_, i.e., rows representing attributes and columns representing attribute vectors, namely, items. By anchoring the items with attribute vectors, proposition experts and measurement experts transform items into measurable forms and then diagnose examinees. In a word, the design of the Q_t_ matrix is the problem of how to match the attribute vectors to achieve a certain predetermined goal.

The CDTDs can be divided into the following aspects based on different dimensions: the dichotomous CDTD (Chiu et al., 2009; Ding et al., 2010) and the polytomous CDTD (Ding et al., 2014a,b,c) according to the scoring methods; Boolean matrix CDTD (Samejima, 1995; Tatsuoka, 1995, 2009; Ding et al., 2011; Cai et al., 2018) and polytomous Q matrix CDTD (Ding et al., 2015; Tu and Cai, 2015) according to the values of elements in the Q_t_ matrix; model-dependent CDTD (Chiu et al., 2009; Kuo et al., 2016) and model-free CDTD (Shao, 2010) according to whether depending on the cognitive diagnostic models (CDM) or not; cognitive diagnostic computerized adaptive testing (CD-CAT) design (Cheng, 2010; Sun et al., 2019) and cognitive diagnostic testing (CDT) design (Henson and Douglas, 2005; Henson et al., 2008; Ding et al., 2011) according to whether personalized diagnostic; independent structure CDTD (Cheng, 2009, 2010; Liu et al., 2016) and dependent structure CDTD (Ding et al., 2011; Kuo et al., 2016) according to cognitive structure, and so on. In fact, almost all CDTDs are multidimensional.

Until present, the studies on the CDTD methods are still relatively weak, and they focus on the following two aspects:

(1) CDTD based on the perfect Q matrix

The so-called “perfect Q matrix” refers to the Q_t_ matrix that makes the ideal response pattern (IRP) and KS correspond one to one. If the Q matrix in tests is a perfect Q matrix, the pattern match ratio (PMR) improves no matter whether the CDTD is either dichotomous or polytomous.

(i) Examples of dichotomous CDTD: For the four attribute hierarchies of Leighton (Leighton et al., 2004), if the Q_t_ matrix is a Boolean matrix, and there is no compensation between the attributes, then the reachable matrix (or it is equivalent classes) acts as the submatrix of Q_t_ which can achieve a one-to-one correspondence between the set of IRPs and the set of KSs. The more reachable matrices in the Q_t_ matrix, the higher the PMR (Ding et al., 2010, 2011). Ding et al. (2010) called such a Q_t_ matrix a sufficient and necessary matrix, i.e., a perfect Q matrix (Cai et al., 2018). The results are similar to those of Chiu et al. (2009), DeCarlo (2011), and Madison and Bradshaw (2015) on independent structures. With the independent structure and four attributes, Samejima (1995) believed that when the Q_t_ matrix was the identity matrix (i.e., the identity matrix of independent structure is a reachable matrix), all of the KSs would not be misjudged. Chiu et al. (2009) also found that the Deterministic Input Noisy “AND” Gate (DINA) model and the Deterministic Input Noisy Output “OR” gate (DINO) model could diagnose all potential attribute mastery patterns when the Q_t_ matrix included the identity matrix. Similar results have been addressed in other studies (DeCarlo, 2011; Madison and Bradshaw, 2015).
(ii) Examples of polytomous CDTD: To achieve the one-to-one correspondence between the set of KSs and the set of IRPs, the rooted tree structure, the independent structure, and the perfect Q matrices of the rhombus structure are introduced under the item score rule that one ideal score is added if mastering one attribute adhering to the item (Ding et al., 2014a). In the initial stage of CD-CAT, each attribute can be diagnosed by using the reachable matrix (Tu et al., 2013). In CD-CAT, the higher the percentage of the examinees is, whose testing items are (or contain) the reachable matrix according to the selection strategy, the higher the PRM is.

(2) CDTD based on the index

The Cognitive Diagnostic Index (CDI) (Henson and Douglas, 2005) and the Attribute-level Discrimination Index (ADI) (Henson et al., 2008) are based on the level of items and attributes for CD. Kuo et al. (2016) indicated that each attribute in the test must be measured at least three times to attain better correct attribute classification, so they proposed modified CDIs and ADIs, namely, MCDI and MADI. The Shannon's entropy (Xu et al., 2003) and posterior-weighted Kullback–Leibler (PWKL) (Cheng, 2009) were introduced in CD-CAT. Cheng (2010) believed that adequate coverage of each attribute could improve the validity of the test scores, and then the attribute-balancing index was proposed. Subsequently, the index was further improved (Yu et al., 2011; Liu et al., 2018; Sun et al., 2019). Adaptive multigroup testing method for cognitive diagnosis (CD-AMGT) (Luo et al., 2018), which selects a group of appropriate items in different diagnosis stages, has the advantages of uniform use of item bank and less time to calculate.

The PMR is the main evaluation index for cognitive diagnostic tests. In CDTD, the pretest evaluation of the PMR is more positive than the posttest evaluation because the designed test can be modified quickly, the designer can make up for possible errors before testing, and material resources and time will be saved. At present, the PMR is the posttest estimation based on the data measured or simulated, so it is impossible to calculate PMR immediately during the design process. Furthermore, it is meaningful to discuss the maximum PMR for the pretest, and the maximum PMR is related to the matching degree between the designed test and the cognitive model, as well as the quality and length of the test.

The rest of the study is organized as follows: First, the TCV used in this study is briefly described. Second, the theoretical proof of the relationships between the TCV and the PMR is introduced in detail. The TCV is then evaluated in a simulation study. The end of the study is the discussion and conclusion.

## Methods

### Cognitive Diagnosis

**Figure 1:**
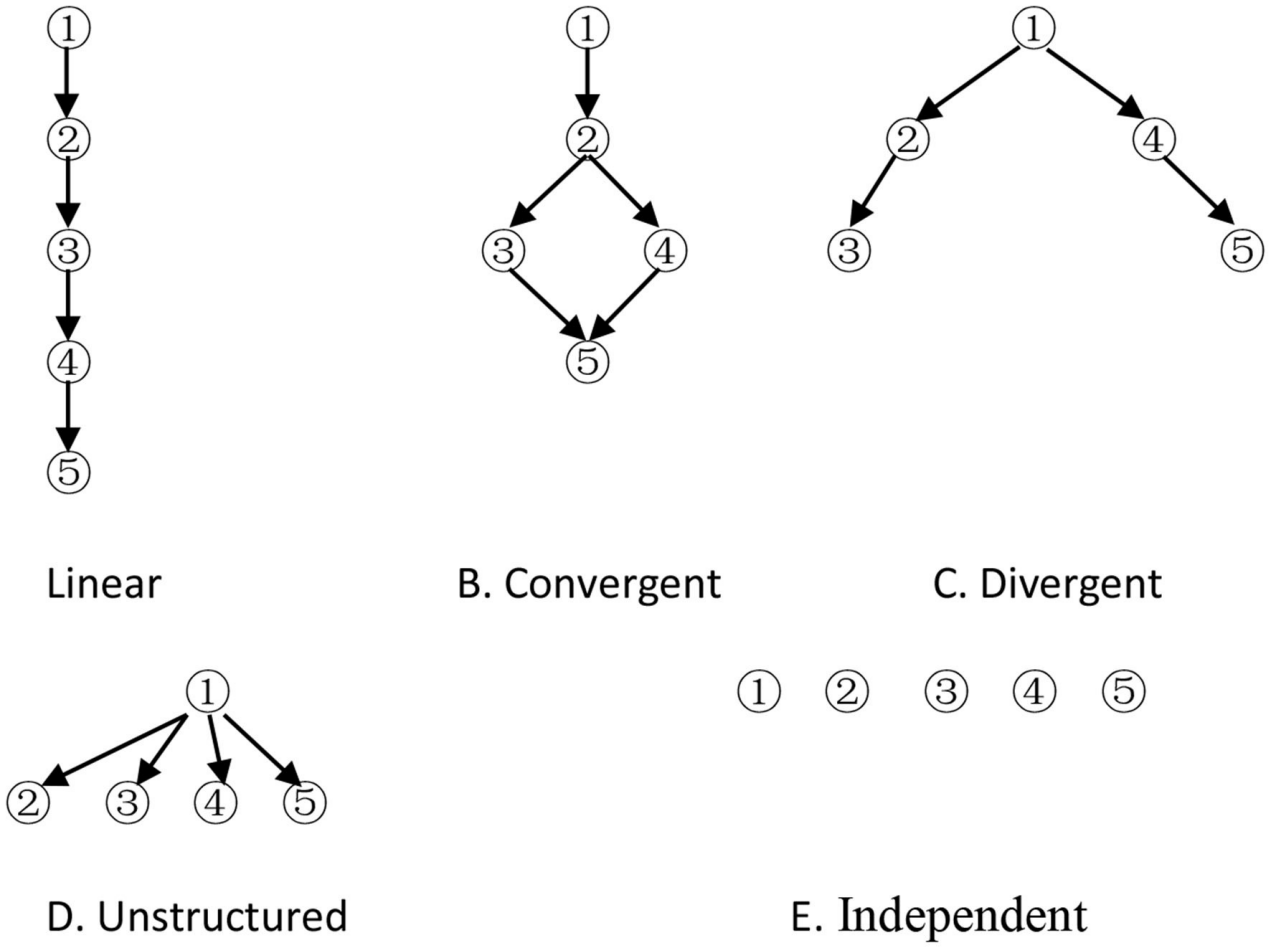
Five different hierarchical structures.

The cognitive model is a prerequisite for CD. It is represented by an attribute hierarchy, which specifies the psychological ordering of the attributes required to solve test items. Attributes are those basic cognitive processes or skills required to solve test items correctly. There are five forms of basic hierarchical structures (Leighton et al., 2004; Cheng, 2010), namely, A, B, C, D and E (Figure 1).

Attribute 1 is considered a prerequisite to other attributes, and attribute 5 depends on some attributes in models except the independent model. The adjacency (A), reachability (R), incidence (Q), and reduced incidence (Q_r_) matrices are specified by Tatsuoka (1995). The columns of the Q_r_ matrix indicate that all possible items must be created to reflect the relationships among the attributes in the hierarchy. The possible latent cognitive states (i.e., KS), which is all the columns of the incidence matrix, possess cognitive attributes that are consistent with the hierarchy (when the hierarchy is based on cognitive considerations), and they apply these attributes systematically (when the hierarchy is based on procedural considerations) (Gierl et al., 2007). Let ${\;q}_{j}{\;=\;\left(q_{j1},q_{j2},\cdots \;,q_{jK\;}\right)}^{T}\left(j\;=\;1,\cdots \;,m\right)$ denote the *j*th dichotomous column vector (i.e., the *j*th category item) of the Q_r_ matrix. All KSs are represented by column vectors: $\alpha_{i}\;=\;{\left(\alpha_{i1},\alpha_{i2},\cdots \;,\alpha_{iK}\right)}^{T}$, where α_*ik*_ = 1(*k* = 1, ⋯ , *K*) indicates that the *i*th category examinee has mastered attribute *k*, and α_*ik*_ = 0 otherwise. *K* is the total number of attributes measured by the test. Let the Q_s_ matrix denote all KSs, in fact, including zero vector ($denoted\;as\;\bar{0},$ i.e., this kind of examinee does not master any attribute) and the Q_r_ matrix for cognitive attribute consistency. Thus, α_*i*_ and *q*_*j*_ are all *K*-dimensional vectors. The Q_t_ matrix consists of some column vectors of the Q_r_ matrix. Based on the cognitive model (including attributes and hierarchy among them), the Q_r_ and Q_s_ matrix can be obtained, that is, all possible items and KSs can be obtained. On the contrary, if the Q_t_ matrix is known, some KSs can be obtained through the augment algorithm (Ding et al., 2008; Yang et al., 2008), and the cognitive model can be derived by comparing the rows (Tatsuoka, 1995). In general, it is impossible for some items (i.e., the Q_t_ matrix) to replace all the items (i.e., the Q_r_ matrix), which express the cognitive structure, so some cognitive structures extracted from the Q_t_ matrix may be inconsistent with the theoretical one.

### The DINA Model

Cognitive diagnostic models have been proposed for many years, including the rule space model (Tatsuoka, 1983), the “Noisy Input Deterministic ‘AND' Gate” (NIDA) model (Maris, 1999), the fusion model (Hartz, 2002), the reduced reparameterized unified model (R-RUM; Hartz, 2002), and the DINA model (Haertel, 1989). The DINA model is completely noncompensatory. The DINA model treats slipping and guessing at the item level. Parameter *s*_*j*_ indicates the probability of “slipping,” and parameter *g*_*j*_ denotes the probability of “guessing.” The item response function, therefore, can be written as follows:

(1) $$\begin{array}{l} \;P\left(X_{ij}\;=\;1|\alpha_{i}\;\right)\;=\;{{{\left(1-s_{j\;}\right)}^{n_{ij\;}}g}_{j\;}}^{1-n_{ij\;}} \end{array}$$

(2) $$\begin{array}{l} n_{ij\;}\;=\;\overset{K}{\underset{k\;=\;1}{\prod }}\alpha_{ik\;}^{q_{jk\;}} \end{array}$$

When *n*_*ij*_ = 1, the *i*th examinee should be able to answer item *j* correctly, unless he/she “slips.” Similarly, when *n*_*ij*_ = 0, the *i*th examinee should not be able to answer item *j* correctly, unless he/she is a lucky guesser (Cheng, 2010).

### Theoretical Construct Validity

Theoretical construct validity (TCV) is used to measure the degree of consistency between the theoretical cognitive model and the cognitive model implied in the Q_t_ matrix (Ding et al., 2012).

**Definition 1** Let {α_1_, α_2_, ⋯ , α_*N*_1__} denote N_1_ KS of the theoretical cognitive model given by experts, {β_1_, β_2_, ⋯ , β_*N*_2__} denote N_2_ KS derived from the Q_t_ matrix, and {γ_1_, γ_2_, ⋯ , γ_*N*_3__} = {β_1_, β_2_, ⋯ , β_*N*_2__}∩{α_1_, α_2_, ⋯ , α_*N*_1__} denote N_3_ KS. when γ_*k*_ = α_*i*_, the TCV for the Q_t_ matrix can be written as follows:

(3) $$\begin{array}{l} \mathrm{TCV}\;=\;\underset{i\;}{\sum }p_{i\;} \end{array}$$

where *p*_*i*_ represents the probability of the *i*th category examinees, that is, the ratio of such examinees whose KS is α_*i*_ in the total population.

In particular, when all KS ratios in the total population are equal, then

(4) $$\mathrm{TCV}\text{ = }\{\begin{array}{c} \frac{{\text{N}}_{\text{3}}\text{+1}}{{\text{N}}_{\text{1}}};\;\bar{0}\notin \left\{\beta_{\text{1}},\beta_{\text{2}},\cdots ,\beta_{{\text{N}}_{\text{2}}}\right\}\\ \frac{{\text{N}}_{\text{3}}}{{\text{N}}_{\text{1}}};\text{ Otherwise} \end{array}$$

In fact, the TCV is a measure of the degree to which the Q_t_ matrix represents the theoretical cognitive model (Ding et al., 2012). The observed response pattern (ORP) and the CDM are necessary for the set of the estimation of KSs of the examinees. The set of IRPs is determined by the set of KSs, the test Q matrix, the element value of the Q_t_ matrix (the dichotomous or the polytomous), the calculation method of the ideal score, the compensation between attributes, and so on. The ORP is related not only to the above mentioned factors but also to the item quality and random factors. Thus, if there is no random factor, the better the item quality, the closer the ORP is to the IRP. Due to the slipping and the guessing in the answering process of examinees, the PMR of the set of KSs estimated by the ORP is not higher than that estimated by the IRP, that is, *PMR*_*ORP*_ ≤ *PMR*_*IRP*_. *The PMR*_*IRP*_ acts as the maximum *PMR*_*ORP*_, and the smaller the slipping and the guessing, the more accurate the KSs based on the ORP. How to get the PMR_IRP_ quickly is an interesting problem.

To clearly solve the interesting problem, a theoretical explanation that makes sense of the complexity is firmly couched within the examples.

**Definition 2** Define the relationship between two attribute vectors α_*i*_ *and q*_*j*_ as α_*i*_ ≥ *q*_*j*_ if and only if α_*ik*_ ≥ *q*_*jk*_, for *k* = 1, 2, …, *K*. Strict inequality between the attribute vectors is involved (i.e., α_*i*_ > *q*_*j*_) if α_*ik*_ > *q*_*jk*_ for at least one *k* (de la Torre, 2011). α_*i*_ ≤ *q*_*j*_ and α_*i*_ < *q*_*j*_ can be defined similarly as mentioned earlier. If the relationship does not exist, then α_*i*_ has nothing to do with *q*_*j*_. The definition of comparison between column vectors also applies to row vectors.

### Examples

The theoretical cognitive model is an independent structure of three attributes, according to the methods suggested by Tatsuoka for calculating the adjacency (A), reachability (R), incidence (Q), and reduced incidence (Q_r_) matrices; then, adding zero vector to the Q_r_ matrix, there are 2^3^ = 8 possible KSs, that is, N_1_ is 8. The Q_s_ matrix is represented by a 3 × 8 matrix as follows:

(5) $$\begin{array}{l} Q_{\text{s}}=\left(\alpha_{1},\alpha_{2},\cdots \;,\alpha_{8}\right)\;=\;\left(\begin{array}{cccccccc} 0 & 1 & 0 & 0 & 1 & 1 & 0 & 1\\ 0 & 0 & 1 & 0 & 1 & 0 & 1 & 1\\ 0 & 0 & 0 & 1 & 0 & 1 & 1 & 1 \end{array}\right) \end{array}$$

where α_*i*_ (*i* = 1, ⋯ , 8) is the *i*th category examinees.

Test items, represented by a 3 × 3 matrix, can be written as follows:

(6) $$\begin{array}{l} Q_{t}\;=\;\left(q_{1},q_{2},q_{3}\right)\;=\;\left(\begin{array}{ccc} 1 & 0 & 1\\ 0 & 1 & 0\\ 0 & 0 & 1 \end{array}\right) \end{array}$$

where *q*_*j*_ is the *j*th item when items are not duplicated, otherwise it represents the *j*th category item.

#### Calculation of TCV

A new matrix, called the $Q_{t\;}^{+\;}matrix,\;$ is made of the *Q*_*t*_ *matrix and the two* new columns. The two new columns based on the augment algorithm (Ding et al., 2008; Yang et al., 2008) are generated from the *Q*_*t*_ *matrix*, $\left(\begin{array}{ccc} 1 & 0 & 1\\ 0 & 1 & 0\\ 0 & 0 & 1 \end{array}\right)|\begin{array}{c} 1\\ 1\\ 0 \end{array}\;|\begin{array}{c} 1\\ 1\\ 1 \end{array}\;$while the non-zero vectors (0, 1, 1)^*T*^ and (0, 0, 1)^*T*^ in the Q_s_ matrix cannot be generated as follows:

(7) $$\begin{array}{l} Q_{t\;}^{+}\;=\;\left(\begin{array}{ccccc} 1 & 0 & 1 & 1 & 1\\ 0 & 1 & 0 & 1 & 1\\ 0 & 0 & 1 & 0 & 1 \end{array}\right) \end{array}$$

Five KSs in the $Q_{t\;}^{+}matrix$ are derived from the *Q*_*t*_ *matrix*, that is, *N*_2_ is 5. There are five same possible latent cognitive states between the theoretical cognitive model and the cognitive model implied in the test design, that is, {γ_1_, γ_2_, γ_3_, γ_4_, γ_5_} = {α_2_, α_3_, α_5_, α_6_, α_8_}, *N*_3_ is 5 (*N*_1_, *N*_2_, and *N*_3_ are the same as Definition 1), when adding zero vector ($\bar{0}={\left(0,0,0\right)}^{T}$).

(1) When the probability distribution of the set of KSs in the total population is discrete uniform, then *TCV* = (5 + 1)/8 = 3/4.
(2) Otherwise, suppose the ratios of all α_*i*_ are 0.1, 0.1, 0, 1, 0.2, 0.1, 0.2, 0.1, 0.1, respectively, *TCV* = 0.1 + 0.1 + 0.1 + 0.2 + 0.1 = 0.6.

#### Calculation of PMR_IRP_

Ideal response (IR) depends on the relationship between α_*i*_ and *q*_*j*_. Let $IR\left(\alpha_{i\;},q_{j}\right)\;=\;{\alpha_{i}}^{o}q{\;}_{j\;}\;=\;\prod_{k\;=\;1}^{K}{\left(\alpha_{ik}\right)}^{q{\;}_{jk\;}}\;=\;1$ denote that the *i*th examinee responses correctly on the *j*th item, and *IR*(α_*i*_, *q*_*j*_) = 0 otherwise. Clearly, *IR*(α_1_, *q*_1_) = *IR*(α_1_, *q*_2_) = *IR*(α_1_, *q*_3_) = 0 due to ${\bar{0}\leq \alpha \;}_{1}<q{\;}_{1}<q{\;}_{3}\;and\;{\bar{0}\leq \alpha \;}_{1}<q{\;}_{2}$; *IR*(α_2_, *q*_1_) = 1, *IR*(α_2_, *q*_2_) = *IR*(α_2_, *q*_3_) = 0 due to *q*__1_ ≤ α 2_ < *q*_3_; and α _2_ *having nothing to do with q*_2_. Similarly, the set of IRPs of the Q_s_ matrix with respect to the *Q*_*t*_ matrix is represented by a 3 × 8 matrix as follows:

(8) $$\begin{array}{l} IRP\;=\;\left(\begin{array}{cccccccc} 0 & 1 & 0 & 0 & 1 & 1 & 0 & 1\\ 0 & 0 & 1 & 0 & 1 & 0 & 1 & 1\\ 0 & 0 & 0 & 0 & 0 & 1 & 0 & 1 \end{array}\right) \end{array}$$

In Equation 8, the row represents the item, and the column represents $\alpha_{i}^{{}^{\prime }}s$ IRP. There are six different IRPs, that is, six KS can be correctly estimated without taking the slipping and the guessing into account. In essence, the estimated five KSs based on five IRPs are the same as vectors in the $Q_{t\;}^{+}\;matrix$ (five different categories), and adding estimated zero vector (because the IRP is zero vector), there are six categories. ${\alpha_{4}\;=\;\left(0,0,1\right)}^{T}$ and ${\alpha_{7}\;=\;\left(0,1,1\right)}^{T}$are the same categories to zero vector ($\alpha_{1}\;=\;\bar{0}$) and $\alpha_{3}{\;=\;\left(0,1,0\right)}^{T}$, respectively; thus, no new categories are generated.

The whole process of dividing the *Q*_s_ matrix can be vividly described as follows: the *Q*_s_ matrix is similar to a line, and five vectors in the $Q_{t\;}^{+}\;matrix$ are similar to five dots that classify the line into six categories in which only one KS can be estimated correctly; therefore, $PMR_{IRP}\;=\;\frac{6}{8}=\frac{3}{4}$.

From calculations 1 and 2, it can be known that TCV = *PMR*_*IRP*_.

Examples of other structures are shown in Table 1.

**Table 1:** The relationships between the theoretical construct validity (TCV) and the *PMR*_*IRP*_ of other structures.

**Theoretical cognitive model**	**Q_**s**_**	**Q_**t**_**	**$Q_{t\;}^{+}$**	**TCV**	**IRP**	**PMR_**IRP**_**
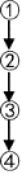	$\left(\begin{array}{ccccc} 0 & 1 & 1 & 1 & 1\\ 0 & 0 & 1 & 1 & 1\\ 0 & 0 & 0 & 1 & 1\\ 0 & 0 & 0 & 0 & 1 \end{array}\right)$	$\left(\begin{array}{ccc} 1 & 1 & 1\\ 0 & 1 & 1\\ 0 & 0 & 1\\ 0 & 0 & 1 \end{array}\right)$	$\left(\begin{array}{ccc} 1 & 1 & 1\\ 0 & 1 & 1\\ 0 & 0 & 1\\ 0 & 0 & 1 \end{array}\right)$	4/5	$\left(\begin{array}{ccccc} 0 & 1 & 1 & 1 & 1\\ 0 & 0 & 1 & 1 & 1\\ 0 & 0 & 0 & 0 & 1 \end{array}\right)$	4/5
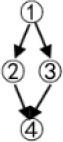	$\left(\begin{array}{cccccc} 0 & 1 & 1 & 1 & 1 & 1\\ 0 & 0 & 1 & 0 & 1 & 1\\ 0 & 0 & 0 & 1 & 1 & 1\\ 0 & 0 & 0 & 0 & 0 & 1 \end{array}\right)$	$\left(\begin{array}{ccc} 1 & 1 & 1\\ 0 & 1 & 1\\ 0 & 0 & 1\\ 0 & 0 & 1 \end{array}\right)$	$\left(\begin{array}{ccc} 1 & 1 & 1\\ 0 & 1 & 1\\ 0 & 0 & 1\\ 0 & 0 & 1 \end{array}\right)$	2/3	$\left(\begin{array}{cccccc} 0 & 1 & 1 & 1 & 1 & 1\\ 0 & 0 & 1 & 0 & 1 & 1\\ 0 & 0 & 0 & 0 & 0 & 1 \end{array}\right)$	2/3
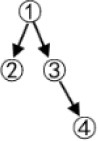	$\left(\begin{array}{ccccccc} 0 & 1 & 1 & 1 & 1 & 1 & 1\\ 0 & 0 & 1 & 0 & 1 & 0 & 1\\ 0 & 0 & 0 & 1 & 1 & 1 & 1\\ 0 & 0 & 0 & 0 & 0 & 1 & 1 \end{array}\right)$	$\left(\begin{array}{ccc} 1 & 1 & 1\\ 1 & 0 & 0\\ 0 & 1 & 1\\ 0 & 0 & 1 \end{array}\right)$	$\left(\begin{array}{ccc} 1 & 1 & 1\\ 1 & 0 & 0\\ 0 & 1 & 1\\ 0 & 0 & 1 \end{array}\right)|\begin{array}{cc} 1 & 1\\ 1 & 1\\ 1 & 1\\ 0 & 1 \end{array}$	6/7	$\left(\begin{array}{ccccccc} 0 & 0 & 1 & 0 & 1 & 0 & 1\\ 0 & 0 & 0 & 1 & 1 & 1 & 1\\ 0 & 0 & 0 & 0 & 0 & 1 & 1 \end{array}\right)$	6/7
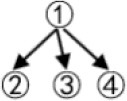	$\left(\begin{array}{ccccccccc} 0 & 1 & 1 & 1 & 1 & 1 & 1 & 1 & 1\\ 0 & 0 & 1 & 0 & 0 & 1 & 1 & 0 & 1\\ 0 & 0 & 0 & 1 & 0 & 1 & 0 & 1 & 1\\ 0 & 0 & 0 & 0 & 1 & 0 & 1 & 1 & 1 \end{array}\right)$	$\left(\begin{array}{ccc} 1 & 1 & 1\\ 1 & 0 & 0\\ 0 & 1 & 1\\ 0 & 0 & 1 \end{array}\right)$	$\left(\begin{array}{ccc} 1 & 1 & 1\\ 1 & 0 & 0\\ 0 & 1 & 1\\ 0 & 0 & 1 \end{array}\right)|\begin{array}{cc} 1 & 1\\ 1 & 1\\ 1 & 1\\ 0 & 1 \end{array}$	2/3	$\left(\begin{array}{ccccccccc} 0 & 0 & 1 & 0 & 0 & 1 & 1 & 0 & 1\\ 0 & 0 & 0 & 1 & 0 & 1 & 0 & 1 & 1\\ 0 & 0 & 0 & 0 & 0 & 0 & 0 & 1 & 1 \end{array}\right)$	2/3

Although structures are different, convergent, or divergent, the result of the relationship between the TCV and the *PMR*_*IRP*_ is the same: the number of vectors of the $Q_{t\;}^{+}matrix$ in the convergent structure was 3, and then the Q_s_ matrix could be classified into four categories; the number of vectors of the $Q_{t\;}^{+}\;matrix$ in the divergent structure was 5 due to two new columns derived from the *Q*_*t*_ *matrix*, and then the Q_s_ matrix could be classified into six categories. For the linear structure and the unstructured, the results are similar.

Notably, all items of the *Q*_*t*_ *matrix* are different because the repetition of items does not increase the “coverage” of the cognitive model by the *Q*_*t*_ *matrix*. Repeated items only reduce random errors; thus, in the following discussion, it is not necessary to consider the repeated items in the *Q*_*t*_ *matrix*.

### Theoretical Derivation of TCV = **PMR**_**IRP**_

Let R denote reachable matrix, *the Q*_*r*_ *matrix* is a set of all possible items that can be written as follows:

(9) $$\begin{array}{l} {\text{Q}}_{r}\;=\;\left\{q_{j}^{{}^{\prime }}|q_{j}^{{}^{\prime }}\;=\;\underset{q\;\in q_{R}\;}{⋃}q\;,q_{R}\subseteq \text{R }\right\} \end{array}$$

In fact, *Q*_*t*_ ⊆ *Q*_*r*_, $Qs\;=\;\left\{\bar{0},Q_{r\;}\;\right\}$.

*For every α*_*i*_ (*i* = 1, ⋯ , *n*) (*except for zero vector*) in the *Q*_s_ matrix, there should be a $q_{j}^{{}^{\prime }}\left(\in Q_{r\;}\right)$ corresponding to it, that is, α ${}_{i\;}=q_{j}^{{}^{\prime }}$.

*Based on the augment algorithm, the*$Q_{t\;}^{+}$*matrix* can be defined as follows:

(10) $$\begin{array}{l} \;{\text{Q}}_{t\;}^{+}\;=\;\left\{q{\;}_{j\;}|q{\;}_{j\;}\;=\;\underset{p\in Q}{\vee }p\;,Q\subseteq Q_{t},j\;=\;1,\ldots ,m\;\right\} \end{array}$$

where V represents the Boolean union operation, *p* ∈ *Q* means that *p* is the item (column) of the *Q* matrix, and *Q* ⊆ *Q*_*t*_ indicates that the *Q* matrix is a subset of the *Q*_*t*_ *matrix* and contains one or more items. New columns of $the\;Q_{t\;}^{+}\;matrix$ can be obtained by the Boolean union of two or more items in the *Q*_*t*_ *matrix*. There are *m* columns in $the\;Q_{t\;}^{+}matrix$, adding zero vector, *m*+1 categories of the KSs are derived from the *Q*_*t*_ *matrix* in total. *n* is the number of the set of KSs derived from the theoretical cognitive model, that is, *n* columns in *the Qs matrix*, so the TCV can be calculated as follows:

(11) $$\begin{array}{l} \text{TCV}=\left(\text{m}+1\right)/\text{n}. \end{array}$$

The maximum lower bound of α_*i*_ can be found in $the\;Q_{t\;}^{+}matrix$ by comparing α_*i*_ with $q{\;}_{j\;}in\;the\;Q_{t\;}^{+}matrix,\;$ and it can be defined as follows:

(12) $$\begin{array}{l} q{\;}_{j^{\prime }}=\max\{q{\;}_{j\;}|q{\;}_{j\;}\leq \alpha_{i},q{\;}_{j\;}\in Q_{t\;}^{+},\alpha_{i}\in Qs,\\ \;i\;=\;1,2,\ldots ,n;j=1,2,\ldots ,m\}\; \end{array}$$

In fact, *j*′ is the subscript of the maximum item, that is, $j^{\prime }\;=\;argmax\;\left\{q{\;}_{j\;}\right\}$, *j*′ ∈ {1, 2, ⋯ , *m* }.

Let $\left\{q{\;}_{j^{\prime }}\right\}$ denote a set of α_*i*_ with the same maximum lower bound $q_{j^{\prime }}$:

(13) $$\begin{array}{l} \left\{q{\;}_{j^{\prime }}\right\}\;=\;\left\{\alpha_{i}|q{\;}_{j\;}\leq \alpha_{i},q{\;}_{j^{\prime }}\;=\;\max\left\{q{\;}_{j\;}\right\}\right\} \end{array}$$

If $q{\;}_{j^{\prime }}$ does not exist, then let $\left\{\bar{0}\right\}\;denote\;\alpha_{i}$ set as follows:

(14) $$\begin{array}{l} \left\{\bar{0}\right\}\;=\;\left\{\alpha_{i}|q{\;}_{j}>\alpha_{i}\;or\;\alpha_{i}\;has\;nothing\;to\;do\;with\;q{\;}_{j}\right\} \end{array}$$

All the $\alpha_{i}^{{}^{\prime }}s$ with the same IRP will be classified into one category by comparing α_*i*_ with all *p* items in the *Q*_*t*_ *matrix*: based on the definition of $q{\;}_{j^{\prime }},\;$if $q{\;}_{j^{\prime }}$ exists, it means that $q{\;}_{j\;}\;=\;\vee_{p\in Q}p\;\leq q{\;}_{j^{\prime }}\leq \alpha_{i}$, so the IRs between α_*i*_ and *p* (*p* ≤ α_*i*_) are 1, that is, $IR\left(\alpha_{i},p\right)\;=\;{\alpha_{i}}^{o}p\;=\;1$, the IRs between α_*i*_ and the rest of *p* in the *Q*_*t*_ *matrix* are 0, that is, *IR* (α_*i*_, *p*) = α_*i*_*p* = 0. Therefore, all the $\alpha_{i}^{{}^{\prime }}s$ in $\left\{q{\;}_{j^{\prime }}\right\}$ have the same IR, and these $\alpha_{i}^{\prime }s\;$ belong to one category. If $q{\;}_{j^{\prime }}$ does not exist, for all *p* items in the *Q*_*t*_ *matrix*, α_*i*_** < ***p* or α_*i*_ *has nothing to do with p*, the IRs between α_*i*_ and *p* is 0, IR is the same with zero vector $\left(\bar{0}\right)$, and thus, these $\alpha_{i}^{\prime }s\;$are the same category as zero vector.

**Proposition 1: All** α_*i*_**s in** **theQ**_**s**_ **matrix** **are classified into** $\left\{q{\;}_{j^{\prime }}\right\}(q{\;}_{j^{\prime }}\in Q_{t\;}^{+})\;or\;\left\{\bar{0}\right\}(i\;=\;1,\ldots ,n;j^{\prime }\;=\;1,\ldots ,m;m\leq n)$.

First, there must be existed a **α**_**i**_ for every $q{\;}_{j^{\prime }}\;in\;the\;Q_{t\;}^{+}matrix$, so that *α_i_*, so $q{\;}_{j^{\prime }}$ is the maximum lower bound of α_*i*_, α_*i*_ is an element of a $set\;\left\{q{\;}_{j^{\prime }}\right\}.$ *m* $\alpha_{i}^{\prime }s\;$ are divided into *m* sets $\left\{q{\;}_{j^{\prime }}\right\}$.

Second, for the remaining *n*-*m* $\alpha_{i}^{\prime }s,$

(1) For every *p* in the *Q*_*t*_ *matrix*, if α_*i*_ < *p* or α_*i*_ *has nothing to do with* *p*, then $q{\;}_{j^{\prime }}$ does not exist, so α_*i*_ belongs to set $\left\{\bar{0}\right\}$;
(2) If *p* ≤ α_*i*_, there must be existed $q{\;}_{j^{\prime }}\;$acted as the maximum lower bound of α_*i*_, so α_*i*_ belongs to set $\left\{q{\;}_{j^{\prime }}\right\}$.

Combining (1) and (2), Proposition 1 is proved.

**Proposition 2: If the number ofq**_**j**_**in the**$Q_{t\;}^{+}$**matrix is*m*, all**$\alpha_{i}^{\prime }$**s in the Q**_**s**_**matrixare classified into*m* + 1 categories**.

From Proposition 1, the conclusion is clearly true, that is, *m* + 1 categories of the set of KSs can be estimated correctly. Thus, $PMR_{IRP}\;=\;\frac{m+1}{n}$. The result of TCV = *PMR*_*IRP*_ shows that the TCV is equal to the PMR estimated by the set of IRPs. For *PMR*_*ORP*_ ≤ *PMR*_*IRP*_ = *TCV*, the TCV is the upper bound of the PMR estimated by the ORP. When *k* is smaller, such as *k* ≤ 5, the TCV can be calculated by pen, otherwise, it is easily derived by using a computer.

## Simulation Study

A simulation study was carried out to evaluate the relationships between the TCV and the PMR.

Five attribute hierarchical structures were studied, namely, independent, linear, convergent, divergent, and unstructured. The number of attributes was set at 4, that is, *K* = 4. The study needed to consider the influence of the distribution of examinees, item attribute vector, and their proportions on the TCV. Two kinds of distribution of the KSs of examinees were discussed as follows: the average distribution (30 persons for every KS) and the normal distribution. In particular, the standard multivariate normal distributions in the independent structure were investigated. The total number of examinees was the same. In contrast, there were six Q_t_ matrices for each structure, items would be selected from the *Q*_*r*_matrix, and its proportions were different. The test length was 20. The descriptive statistics of the examinees and the Q_t_ matrices are reported in Table 2.

**Table 2:** The distributions of examinees and the proportions of items for five different hierarchical structures.

**Attribute structure**	**Distribution of examinees**	**The ratio of examinees**	**The Q_**t**_ matrix**	**The ratio of items**
Linear	The average distribution	Each KS has the same ratio	An item category: (1100)	
			Two item categories: (1000) (1110)	➀1:1 ➁1:3
	The normal distribution	(0000):(1111):(1000):(1110): (1100) = 1:1:2:2:4	Four item categories: (1000, 1100, 1110, 1111)	➀1:1:1:1 ➁2:8:8:2
Convergent	The average distribution	Each KS has the same ratio	Two item category: (1100) (1010)	➀1:1 ➁1:3
	The normal distribution	(0000):(1111): (1000):(1110): (1100):(1010) = 1:1:2:2:7:7	Four item categories: (1000) (1100) (1010) (1110)	➀1:1:1:1 ➁2:8:8:2
			Five item categories: (1000) (1100) (1010) (1110) (1111)	➀1:1:1:1:1➁2:4:8:4:2
Divergent	The average distribution	Each KS has the same ratio	Two item category: (1100) (1011)	➀1:1 ➁1:3
	The normal distribution	(0000):(1111): (1000):(1110): (1100):(1011): (1010) = 10:10:21:21:42:42:64	Four item categories: (1000) (1100) (1010) (1111)	➀1:1:1:1 ➁2:8:8:2
			Six item categories: (1000) (1100) (1010) (1011) (1110) (1111)	➀2:4:4:4:4:2➁2:3:5:5:3:2
Unstructured	The average distribution	Each KS has the same ratio	Four item categories: (1000) (1010) (1101) (1011)	
	The normal distribution	(0000):(1111): (1000):(1011): (1100):(1101): (1010):(1110): (1001) = 16:16:22:22:27:27:43:43:54	Six item categories: (1000) (1100) (1001) (1101) (1011) (1111)	➀1:1:1:1 ➁2:8:8:2 ➀2:4:4:4:4:2➁2:3:5:5:3:2
			Eight item categories: (1000) (1100) (1010) (1001) (1110) (1101) (1011) (1111)	➀2:2:2:4:4:2:2:2➁1::1:3:5:5:3:1:1
Independent	The average distribution	Each KS has the same ratio	Eight item categories: (1000) (0010) (1100) (1001) (1110) (1101) (1011) (1111)	➀2:2:2:4:4:2:2:2 ➁1::1:3:5:5:3:1:1
	The normal distribution	(0000):(1111):(1000):(0111): (0100):(1011):(0010):(1101): (0001):(1110):(1100):(0011): (1010):(0101): (1001):(0110) = 5:5:14:14:24:24:34:34:34:34 :38:38:43:43:48:48	Twelve item categories: (1000) (0100) (0010) (0001) (1100) (1010) (1001) (0101) (0110) (0011) (1110) (1111)	➀1:1:2:2:2:2:2:2:2:2:1:1 ➁1:1:1:1:3:3:3:3:1:1:1:1
	The standard multivariate normal distribution	(0000):(1111):(1000):(0111): (0100):(1011):(0010):(1101): (0001):(1110):(1100):(0011): (1010):(0101): (1001):(0110) = 64:50:77:3:19:3:8:17:0:103: 90:0:28:3:13:2	Fifteen item categories: (1000) (0100) (0010) (0001) (1100) (1010) (1001) (0110) (0101) (0011) (1110) (1101) (1011) (0111) (1111)	➀1:1:1:1:1:1:2:4:2:1:1:1:1:1:1 ➁1:1:1:2:2:2:2:2:2:2:2:2:1:1:1

*The third column is the ratio of the knowledge state (KS); and are two different ratios of items in the Q_t_ matrix; thus, there should be two different Q_t_ matrices*.

To compare the effects of different slips on the TCV and the PMR, the slips were 0.15 and 0.02, respectively. The set of IRPs was obtained by the items of the Q_t_ matrix and the set of KSs of the Q_**s**_ matrix. Let *x* denoted the IR score of an examinee on an item, *r* randomly generated from Uniform (0, 1), if *r* > 1 − *s*, *x* (*x* was dichotomous) would be changed to 1–*x*, and *x* otherwise.

The DINA model and the maximum-likelihood estimation method were used to estimate the KS. Considering the differences in the distribution of examinees, the Q_t_ matrix, and the slips, there were 116 levels in total, and each level was tested 30 times. The final PRM was an average of 30 PMRs.

The PMR index can be defined as follows:

(15) $$\begin{array}{l} PMR\;=\;\frac{\sum_{i\;=\;1}^{N}\alpha_{i-correct\;}}{N} \end{array}$$

where *N* is the number of examinees. α_*i*−*correct*_ = 1 *represents* that the *i*th examinee is estimated correctly.

## Results

Table 3 compares the TCV and the PMR obtained from the linear structure. The first column shows the different distribution of examinees, and the other columns show the results of the different Q_t_ matrices.

**Table 3:** The comparison between the TCV and the PMR_ORP_ of the linear structure.

**Distribution of examinees**	**1 category**	**2 categories**		**4 categories**	
	**➀**	**➀**	**➁**	**➀**	**➁**
The average distribution	TCV = 0.4 PMR = 0.4 (PMR = 0.4)	TCV = 0.6 PMR = 0.5956 (PMR = 0.6)	TCV = 0.6 PMR = 0.5780 (PMR = 0.6)	TCV = 1 PMR = 0.9413 (PMR = 1)	TCV = 1 PMR = 0.7867 (PMR = 0.9827)
The normal distribution	TCV = 0.5 PMR = 0.5 (PMR = 0.5)	TCV = 0.5 PMR = 0.4971 (PMR = 0.5)	TCV = 0.5 PMR = 0.4851 (PMR = 0.5)	TCV = 1 PMR = 0.9164 (PMR = 0.9998)	TCV = 1 PMR = 0.8353 (PMR = 0.9849)

*Values in brackets were the average of PMR when the slip was 0.02, and the following tables were similar*.

Clearly, the TCV was superior: the TCV was uniformly higher than the PMR regardless of the distribution of examinees and the Q_t_ matrices. Although the repetition of items in the Q_t_ matrices, the TCV was not changed when the distribution of examinees and the category of items in the Q_t_ matrices remain unchanged. Therefore, this helped in explaining why repeated items were not necessary to count. As is known to all, the smaller the slip is, the higher the PMR is. But the TCV had nothing to do with the slip, so the smaller the slip, the smaller the gap between the TCV and the PMR. For all the attribute structures, when the TCV was low, the PMR was also low and vice versa. Notably, the more the item categories were, the larger the TCV would be. In particular, if the Q_t_ matrix contained the reachable matrix that could augment all possible item categories, then TCV = 1, regardless of the distribution of examinees. In other words, when the reachable matrix was a submatrix of the Q_t_ matrix, the PMR would be higher than that of the Q_t_ matrix that did not include the reachable matrix if the other conditions were the same.

From Tables 4–7, the data of other structures show the same results as linear. In addition, the lesser the structure, the greater the difference between the TCV and the PMR.

**Table 4:** The comparison between the TCV and the PMR_ORP_ of the convergent structure.

**Distribution of examinees**	**2 categories**		**4 categories**		**5 categories**	
	**➀**	**➁**	**➀**	**➁**	**➀**	**➁**
The average distribution	TCV = 0.6667 PMR = 0.6583 (PMR = 0.6667)	TCV = 0.6667 PMR = 0.6398 (PMR = 0.6667)	TCV = 0.8333 PMR = 0.7726 (PMR = 0.8332)	TCV = 0.8333 PMR = 0.7370 (PMR = 0.8226)	TCV = 1 PMR = 0.8056 (PMR = 0.9935)	TCV = 1 PMR = 0.7600 (PMR = 0.9765)
The normal distribution	TCV = 0.85 PMR = 0.8374 (PMR = 0.85)	TCV = 0.85 PMR = 0.8135 (PMR = 0.8498)	TCV = 0.95 PMR = 0.8748 (PMR = 0.95)	TCV = 0.95 PMR = 0.8954 (PMR = 0.9444)	TCV = 1 PMR = 0.8057 (PMR = 0.9987)	TCV = 1 PMR = 0.8167 (PMR = 0.9898)

**Table 5:** The comparison between the TCV and the PMR_ORP_ of the divergent structure.

**Distribution of examinees**	**2 categories**		**4 categories**		**6 categories**	
	**➀**	**➁**	**➀**	**➁**	**➀**	**➁**
The average distribution	TCV = 0.5714 PMR = 0.5610 (PMR = 0.5714)	TCV = 0.5714 PMR = 0.5316 (PMR = 0.5714)	TCV = 0.8571 PMR = 0.7773 (PMR = 0.8570)	TCV = 0.8571 PMR = 0.7095 (PMR = 0.8329)	TCV = 1 PMR = 0.8083 (PMR = 0.9740)	TCV = 1 PMR = 0.8390 (PMR = 0.9848)
The normal distribution	TCV = 0.4952 PMR = 0.4883 (PMR = 0.4952)	TCV = 0.4952 PMR = 0.4835 (PMR = 0.4952)	TCV = 0.8 PMR = 0.6737 (PMR = 0.7997)	TCV = 0.8 PMR = 0.6694 (PMR = 0.7930)	TCV = 1 PMR = 0.7681 (PMR = 0.9937)	TCV = 1 PMR = 0.8300 (PMR = 0.9962)

**Table 6:** The comparison between the TCV and the PMR_ORP_ of the unstructured structure.

**Distribution of examinees**	**4 categories**		**6 categories**		**8 categories**	
	**➀**	**➁**	**➀**	**➁**	**➀**	**➁**
The average distribution	TCV = 0.6667 PMR = 0.6042 (PMR = 0.6665)	TCV = 0.6667 PMR = 0.5463 (PMR = 0.6548)	TCV = 0.7778 PMR = 0.6446 (PMR = 0.7727)	TCV = 0.7778 PMR = 0.6384 (PMR = 0.7683)	TCV = 1 PMR = 0.7078 (PMR = 0.9774)	TCV = 1 PMR = 0.7363 (PMR = 0.9641)
The normal distribution	TCV = 0.5407 PMR = 0.4846 (PMR = 0.5407)	TCV = 0.5407 PMR = 0.4491 (PMR = 0.5362)	TCV = 0.6815 PMR = 0.5945 (PMR = 0.6780)	TCV = 0.6815 PMR = 0.5483 (PMR = 0.6785)	TCV = 1 PMR = 0.5662 (PMR = 0.9848)	TCV = 1 PMR = 0.7418 (PMR = 0.9815)

**Table 7:** The comparison between the TCV and the PMR_ORP_ of the independent structure.

**Distribution of examinees**	**8 categories**		**12 categories**		**15 categories**	
	**➀**	**➁**	**➀**	**➁**	**➀**	**➁**
The average distribution	TCV = 0.625 PMR = 0.4242 (PMR = 0.6094)	TCV = 0.625 PMR = 0.4515 (PMR = 0.6093)	TCV = 1 PMR = 0.6592 (PMR = 0.9597)	TCV = 1 PMR = 0.6417 (PMR = 0.9737)	TCV = 1 PMR = 0.6110 (PMR = 0.9433)	TCV = 1 PMR = 0.6285 (PMR = 0.9489)
The normal distribution	TCV = 0.5813 PMR = 0.3987 (PMR = 0.5631)	TCV = 0.5813 PMR = 0.4223 (PMR = 0.5628)	TCV = 1 PMR = 0.6953 (PMR = 0.9520)	TCV = 1 PMR = 0.6390 (PMR = 0.9837)	TCV = 1 PMR = 0.6035 (PMR = 0.9472)	TCV = 1 PMR = 0.6438 (PMR = 0.9583)
The standard Multivariate normal distribution	TCV = 0.9438 PMR = 0.5458 (PMR = 0.9014)	TCV = 0.9438 PMR = 0.6421 (PMR = 0.9254)	TCV = 1 PMR = 0.7148 (PMR = 0.9699)	TCV = 1 PMR = 0.7448 (PMR = 0.9792)	TCV = 1 PMR = 0.5848 (PMR = 0.9687)	TCV = 1 PMR = 0.6508 (PMR = 0.9490)

## Discussion and Conclusion

Guided by a cognitive model, the CD can detect how well the examinees have mastered certain knowledge or skills. All CDTDs aim at diagnosing examinees as much as possible, and the main evaluation index is the PMR. The higher the accuracy rate of the KSs, the higher the test construct validity. It is more meaningful to be able to calculate the PMR during CDTD. Tatsuoka (2009, p. 78–79) believed that the sufficient Q matrix can improve the test construct validity. However, how to measure the construct validity? Inspired by the evaluation of the sufficient Q matrix by Tatsuoka (1995, 2009), an evaluation index for cognitive diagnostic test (design) was developed, i.e., TCV, which made up for the defects of Tatsuoka's idea (Tatsuoka, 1995, 2009).

This study proposes a simplified method for predicting the PMR, namely, the TCV method for CD. The TCV intuitive meaning is as follows: the set of KSs is derived from the Q_t_ matrix through the augment algorithm (i.e., this design can inspire some latent cognitive states), and if the probability distribution of the examinees in the population is known, then $TCV\;=\;\underset{j}{\sum }p_{j\;}$. In particular, when the probability distribution of the set of KSs in the total population is discrete uniform, the TCV is equal to the sum, which is the number of categories of the set of KSs derived from the Q_t_ matrix plus 1, divided by the number of categories of the set of KSs in the population. In general, the TCV measures the degree of consistency between the cognitive model derived from matrix Q_t_ and the theoretical cognitive model (Ding et al., 2012).

As the proof and the simulation showed, *PMR*_*ORP*_ ≤ *PMR*_*IRP*_ = *TCV*. Therefore, the TCV can be used to predict the PMR. Notably, the TCV is related to the distribution of examinees and item category, not related to the proportion of items. In other words, when calculating the TCV, repeated items should be treated as one item.

The TCV is numerically equal to the PMR based on the set of IRPs, and the factors that affect the set of IRPs are as follows: the cognitive model (e.g., the number of attributes, attribute hierarchy, and compensation between attributes), the composition of the test matrix (e.g., Boolean matrix and multivalued Q matrix), the item score (e.g., 0–1 score or multilevel score). Whatever has an effect on the set of IRPs influences the TCV. When the test Q matrix (Q_t_) is a Boolean matrix, the score is 0 or 1, and the IR is 1 if and only if α_*i*_ ≥ *q*_*j*_, the TCV is the upper bound of the PMR. The TCV has nothing to do with the CDM (i.e., classification method); therefore, the TCV is calculated by CDM-free. Thus, the conclusion is the same for the DINA model, the AHM (Attribute Hierarchy Method, Gierl et al., 2007) model, the RSM (Rule Space Method, Tatsuoka, 2009) model, and the GDD (Generalized Distance Discrimination, Sun et al., 2011) model.

The number of attributes has an effect on the TCV. For example, independent structure, if the probability distribution of the set of KSs in the total population is equal, different items containing only two attributes are selected, then when the number of attributes *K* is 3, and the TCV is 5/8; when *K* is 4, the TCV is 3/4; and when *K* is 5, the TCV is 27/32. However, under the same conditions, the number of attributes does not affect the conclusion that the TCV is the upper bound of the PMR at all (as shown by the proof). Furthermore, the lower the number of attributes, the higher the PMR. Therefore, the simulation study selected fewer attributes (*K* = 4). Similarly, the smaller the random in the ORP is, the higher the PMR is. To prove that the TCV is the upper bound of the PMR, in the simulation study, the random is relatively small (*s* = 0.02). According to the abovementioned logic, the result that TCV is the upper bound of the PMR is also true when the random is larger.

An interesting question arises as follows: the TCV is not equal to the PMR, why the TCV is useful for predicting the PMR? There are three reasons: First, the most important reason is that the TCV can be obtained during CDTD, which is instructive to adjust selected items at any time and to timely judge the test quality. Second, the TCV is the upper bound of the PMR, the smaller the slip, the smaller the gap between the TCV and the PMR. The TCV does not change with the slip. If the TCV is high, the PMR is also higher; therefore, it is feasible to use the TCV as an index of the PMR to predict the test quality. Third, the TCV is easy to calculate according to the formula.

The TCV can be used not only to predict the PMR but also, more importantly, to detect the defects of CDTD. By using the augment algorithm, the set of KSs can be derived from the Q_t_ matrix, and then, the TCV can be calculated. Under the same conditions, if the TCV value is lower, it means that there are fewer kinds of attribute vectors (i.e., items) of the reachable matrix in the Q_t_ matrix, and thus, the more KSs cannot be accurately estimated. At this time, test designers can modify the test Q matrix (i.e., the Q_t_ matrix) before testing (not posttest evaluation), that is, modify the test (such as filling the columns of the reachable matrix or filling the columns expanded by the reachable matrix through the augment algorithm). Adjusting the selected items according to the TCV value at any time is not only beneficial to evaluate the test quality in time in CDTD but also can save cost and improve efficiency, which has the effect of two times the result with half the effort. This method undoubtedly has great advantages in CDTD.

If the test contains the reachable matrix, the cognitive model derived from the test is consistent with the theoretical cognitive model, and the TCV is 1. At this time, as long as the item quality is good (i.e., the slip is low) and attributes are measured a certain number of times, then the PMR is relatively high. In most cases, however, the PMR is not equal to 1 because the test is short, the quality of the items is poor, or the examinees do not answer carefully. At this time, although the result is rough when the TCV is used to predict the PMR, even so, under the same cases, the test, which contained the reachable matrix (in this case, the Q_t_ matrix is complete Q matrix, Cai et al., 2018), has the higher PMR.

Although this study shows that the TCV method works successfully with CD, it has limitations in several aspects: (1) Since the TCV is determined by the Q_t_ matrix, the Q_t_ matrix must be complete and reliable, which is the premise of using the TCV. In some cases, this condition may be quite harsh. But the RUM model allows the Q_t_ matrix to be incomplete, and the conclusion of this study cannot be applied. Furthermore, the complete and accurate calibration of the Q_t_ matrix is still a very difficult problem. (2) If the score is 0 or 1 and IR is 1 if α_*i*_ ≥ *q*_*j*_, other IR rules are not applicable in this case. Nor does it apply if there is compensation between attributes. (3) Only the dichotomous and non-compensable attributes are considered, a natural question that arises is how to get the TCV when the scoring is polytomous and attributes are compensable. These will be the interesting topics for future studies.

## Data Availability Statement

The raw data supporting the conclusions of this article will be made available by the authors, without undue reservation.

## Author Contributions

XT designed the study, conducted the simulation study, and wrote the manuscript. SD, HD, and MM revised the manuscript. All authors contributed to the article and approved the submitted version.

## Conflict of Interest

The authors declare that the research was conducted in the absence of any commercial or financial relationships that could be construed as a potential conflict of interest.

## Publisher's Note

All claims expressed in this article are solely those of the authors and do not necessarily represent those of their affiliated organizations, or those of the publisher, the editors and the reviewers. Any product that may be evaluated in this article, or claim that may be made by its manufacturer, is not guaranteed or endorsed by the publisher.
